# Creatinine-to-cystatin C ratio and all-cause and cardiovascular mortality in U.S. adults with nonalcoholic fatty liver disease: a nationwide cohort study

**DOI:** 10.3389/fnut.2025.1587757

**Published:** 2025-06-03

**Authors:** Yuanyuan Chen, Bing Yang, Huihui Chen, Jun Chen, Jinmin Cao, Huijie Wang, Chuantie Chen

**Affiliations:** ^1^Department of Liver Diseases, Shenzhen Third People’s Hospital, The Second Affiliated Hospital of Southern University of Science and Technology, National Clinical Research Center for Infectious Diseases, Shenzhen, China; ^2^Department of Gastroenterology, Longgang Central Hospital of Shenzhen, Shenzhen, China; ^3^Guangzhou University of Traditional Chinese Medicine, Guangzhou, China; ^4^Department of Dermatology, Hunan Aerospace Hospital, Changsha, China; ^5^Department of Endoscopy, Shijiazhuang Traditional Chinese Medicine Hospital, Shijiazhuang, China

**Keywords:** creatinine to cystatin C ratio, muscle mass, nonalcoholic fatty liver disease, NHANES, fatty liver index, cohort study, L-shape, mortality

## Abstract

**Background:**

The creatinine-to-cystatin C ratio (CCR) is an emerging marker of muscle mass, which influences the progression of nonalcoholic fatty liver disease (NAFLD). However, the relationship between CCR and long-term all-cause and cardiovascular mortality remains unclear in the US NAFLD population.

**Methods:**

This nationally representative study analyzed data from the National Health and Nutrition Examination Survey (NHANES) 1999–2004, with mortality follow-up through December 31, 2019 via linkage to the National Death Index (NDI). NAFLD was determined using the Fatty Liver Index (FLI), while CCR was calculated as serum creatinine to cystatin C ratio. We employed multivariable Cox proportional hazards models to assess associations between CCR and mortality risk, expressed as hazard ratios (HRs) with 95% confidence intervals (CIs). The analytical approach included Kaplan–Meier survival analysis, restricted cubic spline regression for non-linear relationship assessment, and comprehensive subgroup and sensitivity analyses to evaluate result robustness.

**Results:**

This study included 3,897 participants with NAFLD (53.34% male, mean age 48.98 years), with 1,174 all-cause deaths and 333 cardiovascular deaths over a median follow-up of 206 months. CCR demonstrated significant inverse associations with both all-cause mortality (adjusted HR 0.83; 95% CI 0.78–0.88; *p* < 0.001) and cardiovascular mortality (adjusted HR 0.80; 95% CI 0.73–0.87; *p* < 0.001). In tertile analyses, higher CCR groups showed progressively lower risks, in Model 3(fully adjusted model): all-cause mortality: T2 = 0.65 (0.53, 0.79), T3 = 0.43 (0.32, 0.60), *P* for trend<0.001; cardiovascular mortality: T2 = 0.67 (0.50, 0.89), T3 = 0.34 (0.21, 0.53); *P* for trend<0.001. Restricted cubic spline analysis revealed an L-shaped association between CCR and all-cause mortality (turning point: 11.06), with each unit increase below 11.06 associated with a 36% risk reduction (HR 0.64; 95% CI 0.53–0.77; *p* < 0.001). In contrast, a linear relationship was observed for cardiovascular mortality (*P* for non-linearity = 0.972). Kaplan–Meier analysis confirmed superior survival rates in the highest CCR tertile for both endpoints (log-rank *p* < 0.001), with subgroup and sensitivity analyses affirming the robustness of these results.

**Conclusion:**

In this study of US adults with NAFLD, we identified a significant inverse association between CCR levels and risks of both all-cause and cardiovascular mortality. The stability of this association was confirmed through subgroup and sensitivity analyses, suggesting that CCR may play an important role in long-term prognosis among NAFLD patients.

## 1 Introduction

Nonalcoholic fatty liver disease (NAFLD) is the prevalent chronic liver condition globally, impacting around 24% of the world’s population (1). NAFLD includes a range of liver damage, from simple hepatic steatosis to nonalcoholic steatohepatitis, potentially advancing to cirrhosis, liver failure and hepatocellular carcinoma (2, 3). It is also associated with extrahepatic metabolic disorders, including cardiovascular events and type 2 diabetes mellitus (4). Notably, patients with hepatic steatosis accompanied by advanced liver fibrosis exhibit a significantly elevated risk of both all-cause mortality (5, 6) and cardiovascular disease mortality (7, 8). The increased cardiovascular risk in these patients is thought to be driven by involving interrelated pathways of insulin resistance, oxidative stress, chronic inflammation, endothelial dysfunction, gut microbiota alterations, and dysregulated lipid metabolism (9–12). The newly proposed metabolic dysfunction-associated fatty liver disease (MAFLD) (13) and metabolic dysfunction-associated steatotic liver disease (MASLD) (14) concepts place greater emphasis on the metabolic nature of this disease. However, given their substantial population overlap with NAFLD, this study maintains the traditional NAFLD diagnostic criteria.

Low muscle mass has been established as a significant risk factor for the progression of NAFLD (15). Creatinine, primarily derived from muscle tissue, particularly skeletal muscle (16), is a byproduct of muscle metabolism and is almost entirely excreted by the kidneys. However, creatinine production is not solely dependent on muscle mass but is also influenced by various factors such as age, sex, ethnicity, and dietary habits (e.g., meat intake), especially in elderly individuals or patients with impaired renal function (17). CysC is a small, non-ionic protein synthesized by all nucleated cells. It is filtered by the glomeruli and then fully reabsorbed and metabolized by proximal tubular cells, making it less affected by muscle mass (18). CCR was calculated as serum creatinine to cystatin C ratio. It is considered a marker for muscle mass and a surrogate indicator of sarcopenia (19–21).

Tetssuka et al. (22) conducted a study suggested that the serum CCR may be an indicator for evaluating amyotrophic lateral sclerosis severity. Kashani et al. (18) confirmed the relationship between CCR and muscle mass, introducing it as sarcopenia index in 2017. Currently, CCR has been widely proposed as a biomarker for muscle mass and a surrogate indicator for sarcopenia (19–21). A Japanese study involving 641 participants demonstrated a significant association between CCR and muscle mass as well as strength with NAFLD patients (15). Moreover, research suggests that CCR is more suitable than muscle mass measurement alone for evaluating visceral fat mass adjusted for muscle mass (23). It exhibits high diagnostic accuracy in assessing muscle mass and sarcopenia in conditions such as diabetes (21), and cancer (24). A retrospective cohort study revealed that elevated serum CCR ratios were significantly associated with reduced all-cause mortality at 30 and 90 days in patients undergoing continuous kidney replacement therapy (25). In a U.S. adult cohort, CCR was inversely correlated with all-cause mortality, cardiovascular mortality (26, 27), and cancer mortality (26). However, research on the relationship between CCR and cardiovascular or all-cause mortality with NAFLD populations remains unexplored.

Among individuals with NAFLD, long-term follow-up studies on the association between CCR and all-cause and cardiovascular mortality remain limited. Leveraging weighted data from NHANES 1999–2004 and NDI database, this study utilizes a nationally representative sample to investigate the relationship between CCR and long-term risks of all-cause and cardiovascular mortality in individuals with NAFLD.

## 2 Materials and methods

### 2.1 Study population

The data analyzed were sourced from the NHANES database, a national program that assesses the health and nutritional status of U.S. adults and children using a rigorous multi-stage probabilistic sampling method to ensure national representativeness (28). The Centers for Disease Control and Prevention’s National Center for Health Statistics research ethics review board approved the NHANES study protocol, and participants provided written informed consent at enrollment (the website is https://www.cdc.gov/nchs/nhanes/about/erb.html). Ethical approval and consent were not required as this study was based on publicly available de-identified data. Our secondary analysis adheres to the Strengthening the Reporting of Observational Studies in Epidemiology (STROBE guidelines) for cohort studies. Comprehensive details about the NHANES survey can be accessed publicly at https://wwwn.cdc.gov/nchs/nhanes.

**Figure 1:**
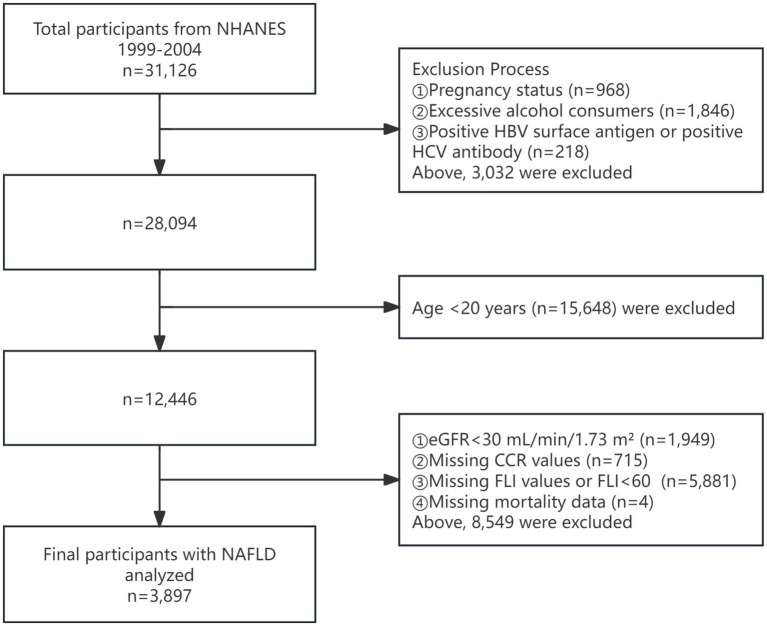
Flowchart of the enrolled participants. NHANES, National Health and Nutrition Examination Survey; eGFR, estimated glomerular filtration rate; CCR, creatinine to cystatin C ratio; FLI, fat liver index; NAFLD, nonalcoholic fatty liver disease.

In this cohort study, the data from three survey cycles (1999–2004) were used because the CysC information was available, and there were 31,126 participants in the cohort. We excluded subjects with pregnancy status (*n* = 968), excessive alcohol consumers (defined as having 5 or more drinks daily) (*n* = 1,846), positive HBV surface antigen or HCV antibody (*n* = 218). In addition, we also excluded participants under 20 years old (*n* = 15,648). Moreover, we excluded individuals with eGFR less than 30 mL/min/1.73m^2^ (*n* = 1,949), missing values for CCR (*n* = 715), missing values for FLI or FLI < 60 (*n* = 5,881), missing values for mortality (*n* = 4). A total of 3,897 subjects with NAFLD participated in this study. Figure 1 illustrates the complete data selection process.

### 2.2 Definition of NAFLD

Although histopathological biopsy remains the gold standard for assessing hepatic steatosis and fibrosis, FLI is gaining widespread use as a non-invasive method in clinical practice. FLI is a straightforward score, ranging from 0 to 100, derived from BMI, waist circumference, triglycerides, and gamma-glutamyl transferase levels to assess fatty liver probability. FLI, with a value of ≥ 60, is used to diagnose NAFLD in the absence of chronic liver disease and significant alcohol consumption (29). This non-invasive diagnostic tool is gaining wider adoption.

FLI is calculated using the formula below (30):


$$\begin{array}{l} \mathrm{FLI}=(e^{0.953*\ln(\mathrm{TG})+0.139*\mathrm{BMI}+0.718*\ln(\mathrm{GGT})+0.053*\mathrm{WC}-15.745})/\\ (1+e^{\begin{array}{l} 0.953*\ln(\mathrm{TG})+0.139*\mathrm{BMI}+0.718*\ln(\mathrm{GGT})+\\ 0.053*\mathrm{WC}-15.745 \end{array}})*100 \end{array}$$

### 2.3 Definition of CCR

Serum creatinine levels in the NHANES datasets were assessed using the kinetic alkaline picrate method, known as the Jaffe reaction. The methodology for measuring serum creatinine in NHANES is documented in other sources. According to NHANES analytical guidelines, creatinine values from 1999 to 2000 require adjustment using Deming regression (standard creatinine = 1.013 * NHANES creatinine + 0.147), whereas no such correction is necessary for NHANES 2001–2002 and 2003–2004 (31). Serum CysC levels were determined using a CysC immunoassay on a Siemens Dimension Vista 1,500 automated multi-channel analyzer (Siemens Healthcare Diagnostics). The detection range spans from 0.23 mg/L to 8.00 mg/L. CCR is calculated by dividing serum creatinine (mg/dL) by cystatin C (mg/L) and multiplying by 10 (26). Initially, CCR was analyzed as a continuous variable and subsequently categorized into tertiles for further analysis.

### 2.4 Ascertainment of mortality

The mortality data were obtained from NDI maintained by the Centers for Disease Control and Prevention (accessible at https://www.cdc.gov/nchs/data-linkage/mortality-public.htm). Participant follow-up duration was calculated from study enrollment until either the occurrence of death or the cutoff date of December 31, 2019, which represents the most recent update in the NDI records. Cardiovascular-related fatalities were classified using specific diagnostic codes (I00-I09, I11, I13, and I20-I51) as defined by the Tenth Revision of the International Classification of Diseases (ICD-10) system (32).

### 2.5 Assessment of covariates

Gender, age, race, education level, marital status, income to poverty ratio (PIR), moderate activity, smoke status, hypertension, diabetes, coronary heart disease, cancer and BMI were sourced from NHANES survey questionnaires. Age was treated as a continuous variable, while gender and race were considered categorical variables. Race categories included as non-Hispanic White, non-Hispanic Black, Mexican American and other. Education level was categorized as less than high school, high school, university and above. Marital status was categorized as married or living with partner, never married, or other. PIR was categorized as low (PIR ≤ 1.3), medium (PIR > 1.3 to 3.5) or high (PIR > 3.5). Moderate activity was defined as activities that participants engage in for at least 10 min continuously and result in minor increases in breathing or heart rate. Smoke status, was defined as history of smoking at least 100 cigarettes. Hypertension was defined as a self-reported history of hypertension. Diabetes was defined as a self-reported history of diabetes. Coronary heart disease was defined as a self-reported history of coronary heart disease. Cancer was defined as a self-reported history of cancer. BMI was calculated as weight in kilograms divided by height (m) squared. The estimated glomerular filtration rate (eGFR) was calculated using the Chronic Kidney Disease Epidemiology Collaboration Scr equation (33). Glycosylated hemoglobin A1C (HbA1c), uric acid (UA), total cholesterol (TC), high-density lipoprotein cholesterol (HDL), and triglyceride (TG) were obtained from laboratory test results.

### 2.6 Statistical analysis

In our study, analyses followed the National Center for Health Statistics’ stratification and weighting guidelines for NHANES (34). For the weighted analyses of NHANES 1999–2000 and 2001–2002 data, a four-year sample weight (WTSSCB4Y) set was used. For the 2003–2004 data, the sample weight (WTSSCB2Y) set was used. The sampling weights for 1999–2004 were calculated as follows: 1999–2002 weights are 2/3 × WTSSCB4Y, otherwise 1/3 × 2003–2004. Missing values in covariates were addressed using a multivariate single imputation method. This approach utilized an iterative imputer, with a Bayesian Ridge model serving as the estimator in each step of the round-robin imputation process (35). Multicollinearity was assessed using variance inflation factor, with a threshold of 5 indicating potential collinearity. After excluding participants with missing covariates, sensitivity analyses were performed on the final study population. Continuous variables with a normal distribution are presented as mean ± standard deviation (SD), those with a non-normal distribution as interquartile range (IQR), and categorical variables as percentages (%). CCR was divided into tertiles from lowest (T1) to highest (T3). Categorical data differences among the three subgroups were assessed using chi-squared tests. One-way ANOVA was used for normally distributed data, while the Kruskal-Wallis test was applied for data that did not follow a normal distribution. A restricted cubic spline (RCS) with three knots was used to illustrate the potential nonlinear relationship between the CCR and both all-cause and cardiovascular mortality with NAFLD patients. Survey-weighted Cox regression analysis was used to evaluate the relationship between CCR and both all-cause and cardiovascular mortality in patients with NAFLD. Three models were developed with increasing levels of adjustment for potential outcome confounders: Model 1 was unadjusted; Model 2 accounted for gender, age and race; model 3 was adjusted for gender, age, race, education level, marital status, PIR, moderate activity, smoke status, hypertension, diabetes, coronary heart disease, cancer, BMI, eGFR, HbA1c, UA, HDL, TG, and TC. Survival outcome probabilities were estimated using the Kaplan–Meier method and compared via the log-rank test. The relationship between CCR and mortality was examined across subgroups defined by gender, age, hypertension, diabetes, coronary heart disease, cancer and eGFR, and their interactions were explored. Statistical analyses were performed using R software (version 4.2.2; http://www.r-project.org). All tests were two-tailed and *p* values less than 0.05 were considered statistically significant.

## 3 Results

### 3.1 Baseline characteristics of participants

Table 1 presents the demographic, comorbidity, socioeconomic, and laboratory data of the 3,897 participants, representing 62,884,461 NAFLD patients in the U.S. The cohort consisted of 2,004 males (53.34%) and 1,893 females (46.66%), with a median age of 48.98 years. Participants were stratified into three groups based on CCR tertiles: T1 (4.053–9.821), T2 (9.827–11.887), T3 (11.887–61.35). Compared to the lowest CCR tertile (T1), participants in higher CCR tertiles were more likely to be male, younger, have higher education levels, better PIR, higher moderate physical activity levels and no history of smoking. Additionally, they exhibited a lower prevalence of hypertension, diabetes, coronary heart disease, and cancer, along with better metabolic profiles, including lower BMI, higher eGFR, lower HbA1c, and higher HDL levels (all *p* < 0.05). Notably, the highest CCR tertile (T3) was predominantly male (82.16%), with significant socioeconomic advantages, including higher education levels (T3: 60.46% vs. T1: 41.16%) and better income status (T3: 52.59% vs. T1: 31.74%) (all *p* < 0.001).

**Table 1:** Characteristics of the study population based on CCR.

Variables	Overall (4.053–61.35, *n* = 3,897)	Tertile of CCR			*P* value
		T1 (4.053–9.821, *n* = 1,297)	T2 (9.827–11.887, *n* = 1,299)	T3 (11.887–61.350, *n* = 1,301)	
Gender, n (%)					<0.001
Male	2,004 (53.34%)	257 (18.83%)	683 (51.96%)	1,064 (82.16%)	
Female	1,893 (46.66%)	1,040 (81.17%)	616 (48.04%)	237 (17.84%)	
Age (years)	48.98 (0.35)	55.01 (0.58)	48.64 (0.60)	44.49 (0.37)	<0.001
Race, n (%)					<0.001
Non-Hispanic White	1,956 (72.39%)	678 (76.57%)	669 (72.40%)	609 (69.03%)	
Non-Hispanic Black	694 (10.85%)	121 (6.21%)	217 (10.12%)	356 (15.22%)	
Mexican American	992 (7.92%)	415 (8.40%)	328 (8.06%)	249 (7.40%)	
Other	255 (8.85%)	83 (8.82%)	85 (9.42%)	87 (8.35%)	
Education level, n (%)					<0.001
Less than high school	639 (6.82%)	313 (10.05%)	204 (6.84%)	122 (4.21%)	
High school	1,627 (41.96%)	566 (48.79%)	563 (43.25%)	498 (35.33%)	
University and above	1,631 (51.22%)	418 (41.16%)	532 (49.91%)	681 (60.46%)	
Marital status, n (%)					<0.001
Married/Living with Partner	2,541 (67.45%)	759 (60.81%)	860 (67.26%)	922 (72.93%)	
Never married/other	1,356 (32.55%)	538 (39.19%)	439 (32.74%)	379 (27.07%)	
PIR, n (%)					<0.001
Low income	1,079 (20.86%)	463 (28.24%)	355 (21.04%)	261 (14.81%)	
Medium income	1,526 (36.46%)	519 (40.01%)	516 (37.57%)	491 (32.60%)	
High income	1,292 (42.68%)	315 (31.74%)	428 (41.40%)	549 (52.59%)	
Moderate activity, n (%)					<0.001
Yes	1,675 (48.82%)	438 (39.26%)	567 (48.98%)	670 (56.30%)	
No	2,222 (51.18%)	859 (60.74%)	732 (51.02%)	631 (43.70%)	
Smoke status, n (%)					0.007
Yes	1,875 (48.56%)	613 (51.66%)	652 (50.54%)	610 (44.25%)	
No	2,022 (51.44%)	684 (48.34%)	647 (49.46%)	691 (55.75%)	
Hypertension, n (%)					<0.001
Yes	1,679 (39.70%)	661 (49.66%)	535 (37.34%)	483 (33.93%)	
No	2,218 (60.30%)	636 (50.34%)	764 (62.66%)	818 (66.07%)	
Diabetes, n (%)					<0.001
Yes	579 (11.14%)	236 (14.12%)	211 (12.53%)	132 (7.47%)	
No	3,318 (88.86%)	1,061 (85.88%)	1,088 (87.47%)	1,169 (92.53%)	
Coronary heart disease, n (%)					0.012
Yes	206 (4.51%)	78 (5.53%)	72 (5.27%)	56 (2.99%)	
No	3,691 (95.49%)	1,219 (94.47%)	1,227 (94.73%)	1,245 (97.01%)	
Cancer, n (%)					<0.001
Yes	340 (8.25%)	146 (12.07%)	123 (8.24%)	71 (5.20%)	
No	3,557 (91.75%)	1,151 (87.93%)	1,176 (91.76%)	1,230 (94.80%)	
BMI (kg/m^2^)	33.43 (0.17)	35.11 (0.32)	33.57 (0.19)	31.95 (0.19)	<0.001
eGFR(mL/min/1.73 m^2^)	91.15 (0.45)	90.76 (0.82)	93.66 (0.69)	89.15 (0.61)	<0.001
HbA1c (%)	5.73 (0.02)	5.86 (0.03)	5.76 (0.04)	5.61 (0.03)	<0.001
UA (mg/dl)	5.94 (0.04)	5.71 (0.06)	5.81 (0.06)	6.26 (0.05)	<0.001
TC(mg/dl)	211.98 (1.00)	212.80 (1.97)	208.82 (1.55)	214.24 (1.54)	0.037
HDL (mg/dl)	45.46 (0.29)	47.02 (0.42)	45.38 (0.41)	44.29 (0.41)	<0.001
TG (mg/dl)	197.02 (4.15)	199.77 (7.18)	191.56 (4.86)	199.87 (7.09)	0.60

CCR, creatinine to cystatin C ratio; T, tertile; BMI, body mass index; eGFR, estimated glomerular filtration rate; HbA1c, glycosylated hemoglobin type A1C; UA, uric acid; TC, total cholesterol; HDL, hdl-cholesterol; TG, triglycerides; Note: The sample sizes presented in Table are unweighted counts reflecting the actual number of observations. All other results reported in this table are based on weighted data to account for the complex survey design and to ensure representativeness of the study population. The weighting procedure adjusts for potential sampling biases and non-response, allowing for more accurate estimates that are generalizable to the target population.

### 3.2 Associations of the CCR with all-cause mortality

During a median follow-up of 206 months (IQR: 187–225), 1,174 all-cause deaths were recorded. Multivariable Cox regression analyses revealed a significant inverse association between CCR and all-cause mortality. In the unadjusted model (Model 1), each unit increase in CCR was associated with a 21% reduction in all-cause mortality risk (HR: 0.79, 95% CI: 0.76–0.83, *p* < 0.001) (Table 2). This protective association remained robust after adjusting for demographic factors (Model 2: HR: 0.85, 95% CI: 0.80–0.89, *p* < 0.001) and further adjustment for socioeconomic, lifestyle, and clinical variables (Model 3: HR: 0.83, 95% CI: 0.78–0.88, *p* < 0.001) (Table 2).

**Table 2:** The relationships between CCR and mortality with NAFLD.

Variable	Total	Events	Model 1		Model 2		Model 3	
			HR (95%CI)	*P* value	HR (95%CI)	*P* value	HR (95%CI)	*P* value
All-cause mortality								
CCR	3,897	1,174	0.79 (0.76, 0.83)	<0.001	0.85 (0.80, 0.89)	<0.001	0.83 (0.78, 0.88)	<0.001
CCR tertile								
T1	1,297	545	1(Ref)		1(Ref)		1(Ref)	
T2	1,299	379	0.58 (0.49, 0.68)	<0.001	0.69 (0.57, 0.84)	<0.001	0.65 (0.53, 0.79)	<0.001
T3	1,301	250	0.32 (0.25, 0.40)	<0.001	0.47 (0.35, 0.64)	<0.001	0.43 (0.32, 0.60)	<0.001
*P* for trend				<0.001		<0.001		<0.001
Cardiovascular mortality								
CCR	3,897	333	0.78 (0.73, 0.83)	<0.001	0.80 (0.73, 0.88)	<0.001	0.80 (0.73, 0.87)	<0.001
CCR tertile								
T1	1,297	155	1(Ref)		1(Ref)		1(Ref)	
T2	1,299	106	0.64 (0.51, 0.80)	<0.001	0.71 (0.54, 0.93)	0.012	0.67 (0.50, 0.89)	0.006
T3	1,301	72	0.28 (0.20, 0.38)	<0.001	0.36 (0.24, 0.56)	<0.001	0.34 (0.21, 0.53)	<0.001
*P* for trend				<0.001		<0.001		<0.001

Model 1 unadjusted. Model 2 adjusted for gender, age and race. Model 3 further adjusted for education level, marital status, PIR, moderate activity, smoke status, hypertension, diabetes, coronary heart disease, cancer, BMI, eGFR, HbA1c, UA, HDL, TG and TC based on Model 2. CCR, creatinine to cystatin C ratio; NAFLD, nonalcoholic fatty liver disease; T, tertile; HR, hazard ratio; CI, confidence interval; Ref, reference; PIR, income to poverty ratio; BMI, body mass index; eGFR, estimated glomerular filtration rate; HbA1c, glycosylated hemoglobin type A1C; UA, uric acid; HDL, hdl-cholesterol; TG, triglycerides; TC, total cholesterol.

When CCR was analyzed by tertiles, participants in higher tertiles exhibited progressively lower risks of all-cause mortality compared to those in the lowest tertile (T1). In the fully adjusted model (Model 3), as compared to the T1 group, the hazard ratios (HRs) for T2, and T3 were 0.65 (0.53, 0.79) and 0.43 (0.32, 0.60), respectively (*P* for trend < 0.001) (Table 2). These results suggest a dose–response relationship, with the highest CCR tertile (T3) associated with a 57% reduction in all-cause mortality risk compared to T1.

**Figure 2:**
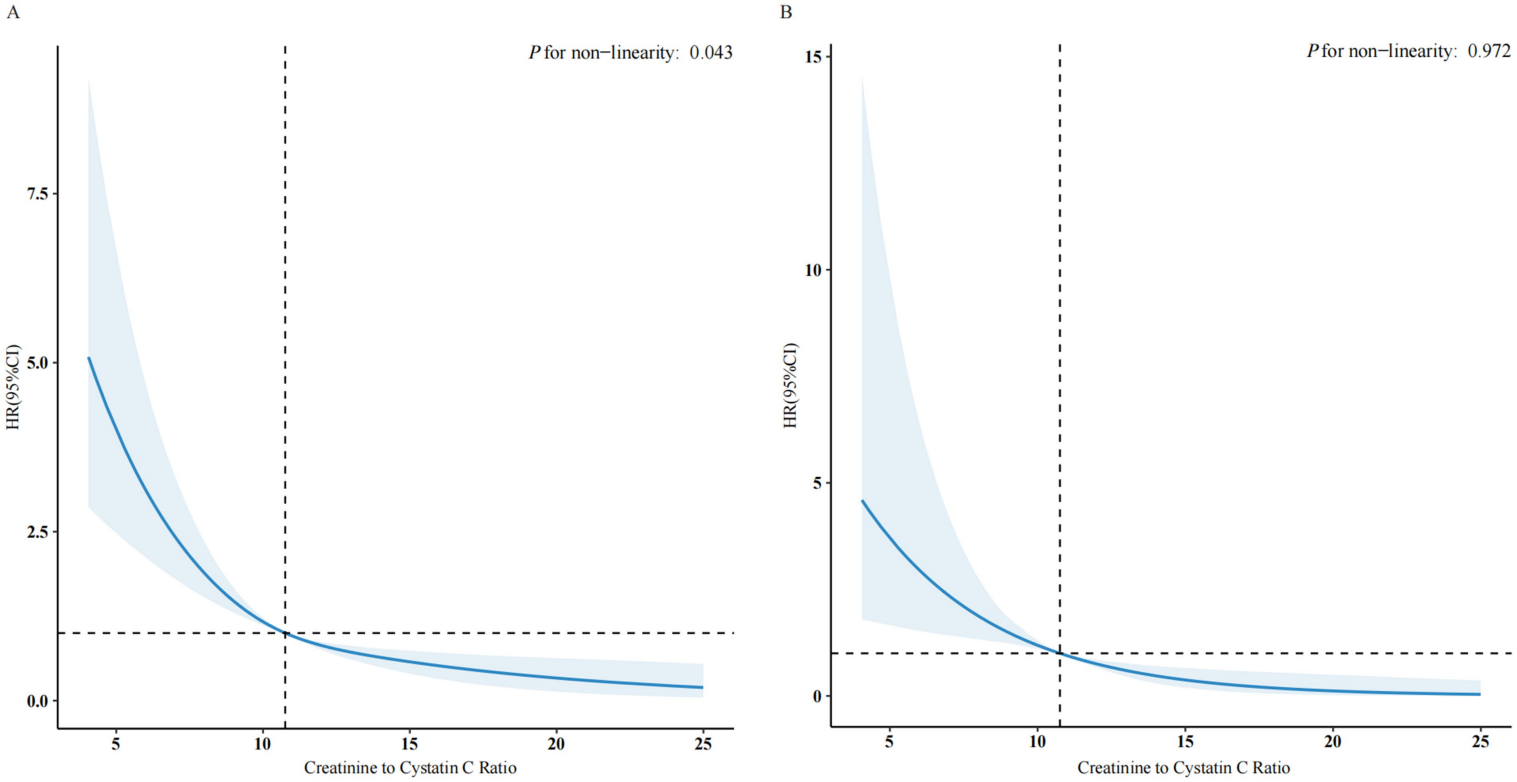
The association of CCR with all-cause **(A)** and cardiovascular mortality **(B)** among NAFLD visualized by restricted cubic spline. HRs were adjusted for gender, age, race, education level, marital status, PIR, moderate activity, smoke status, hypertension, diabetes, coronary heart disease, cancer, BMI, eGFR, HbA1c, UA, HDL, TG and TC. Only 99.9% of the data is shown. CCR, creatinine to cystatin C ratio; NAFLD, nonalcoholic fatty liver disease; HR, hazard ratio; CI, confidence interval; PIR, income to poverty ratio; BMI, body mass index; eGFR, estimated glomerular filtration rate; HbA1c, glycosylated hemoglobin type A1C; UA, uric acid; HDL, hdl-cholesterol; TG, triglycerides; TC, total cholesterol.

RCS analysis further elucidated the relationship between CCR and all-cause mortality, revealing a nonlinear, L-shaped association (*P* for non-linearity = 0.043) (Figure 2A). The risk of all-cause mortality decreased sharply with increasing CCR levels up to a threshold of 11.06, after which the association plateaued. Threshold analysis confirmed this critical turning point, with participants below the threshold (CCR < 11.06) experiencing a 36% reduction in mortality risk per unit increase in CCR (HR: 0.64, 95% CI: 0.53–0.77, *p* < 0.001) (Table 3). In contrast, no significant association was observed above the threshold (CCR ≥ 11.06; HR: 0.87, 95% CI: 0.71–1.06, *p* = 0.15) (Table 3).

**Table 3:** Threshold effect analysis of the relationship of CCR with all-cause mortality among NAFLD.

CCR	Adjusted Model	
	HR (95% CI)	*P* value
< 11.06	0.64 (0.53, 0.77)	<0.001
≥ 11.06	0.87 (0.71, 1.06)	0.150
*P* for log-likelihood ratio		<0.001

Adjusted for gender, age, race, education level, marital status, PIR, moderate activity, smoke status, hypertension, diabetes, coronary heart disease, cancer, BMI, eGFR, HbA1c, UA, HDL, TG and TC. Only 99.9% of the data is shown. CCR, creatinine to cystatin C ratio; NAFLD, nonalcoholic fatty liver disease; HR, hazard ratio; CI, confidence interval; PIR, income to poverty ratio; BMI, body mass index; eGFR, estimated glomerular filtration rate; HbA1c, glycosylated hemoglobin type A1C; UA, uric acid; HDL, hdl-cholesterol; TG, triglycerides; TC, total cholesterol.

### 3.3 Associations of the CCR with cardiovascular mortality

During the same follow-up period, 333 cardiovascular deaths were recorded. Similar to all-cause mortality, higher CCR levels were associated with a reduced risk of cardiovascular mortality. In the unadjusted model (Model 1), each unit increase in CCR was associated with a 22% reduction in cardiovascular mortality risk (HR: 0.78, 95% CI: 0.73–0.83, *p* < 0.001) (Table 2). This association persisted after adjusting for confounders (Model 2: HR: 0.80, 95% CI: 0.73–0.88, *p* < 0.001; Model 3: HR: 0.80, 95% CI: 0.73–0.87, *p* < 0.001) (Table 2). These results suggest a significant negative correlation between higher CCR levels and lower cardiovascular mortality risk.

Based on the CCR tertile groups, we found that as the CCR by tertiles increased, the risk of cardiovascular mortality significantly decreased (*P* for trend < 0.001). After adjusting for confounding factors in Model 3, as compared to the T1 group, the HRs for T2 and T3 groups compared with T1 were 0.67 (0.50, 0.89) and 0.34 (0.21, 0.53), respectively (Table 2). This indicates that individuals with higher CCR levels have significantly lower cardiovascular mortality risks compared to those with lower CCR levels.

RCS analysis revealed a linear, inverse relationship between CCR and cardiovascular mortality (*P* for nonlinearity = 0.972) (Figure 2B). This indicates that increasing CCR is directly associated with a reduced risk of cardiovascular mortality, without a clear turning point.

### 3.4 Survival analysis

**Figure 3:**
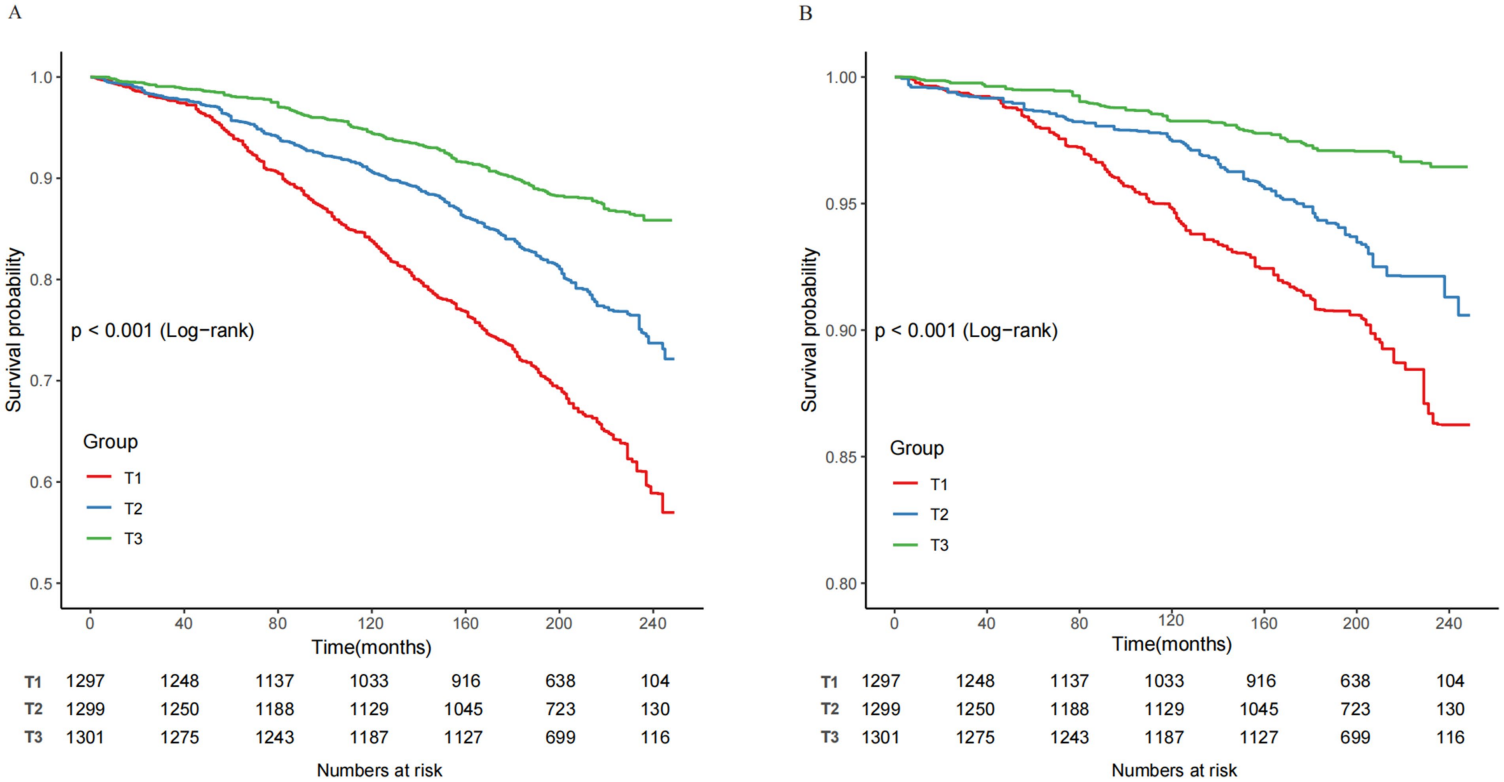
Kaplan–Meier curves of the survival rate and the number of at-risk NAFLD patients with CCR tertiles. **(A)** All-cause mortality. **(B)** Cardiovascular mortality. CCR, creatinine to cystatin C ratio; NAFLD, nonalcoholic fatty liver disease; T, tertile.

Kaplan–Meier survival curves indicated a significant link between elevated CCR levels and enhanced survival outcomes. Participants in the highest CCR tertile (T3) demonstrated significantly improved survival rates for both all-cause and cardiovascular mortality compared to those in lower tertiles (log-rank *p* < 0.001) as shown in Figures 3A,B.

### 3.5 Subgroup and sensitivity analyses

**Figure 4:**
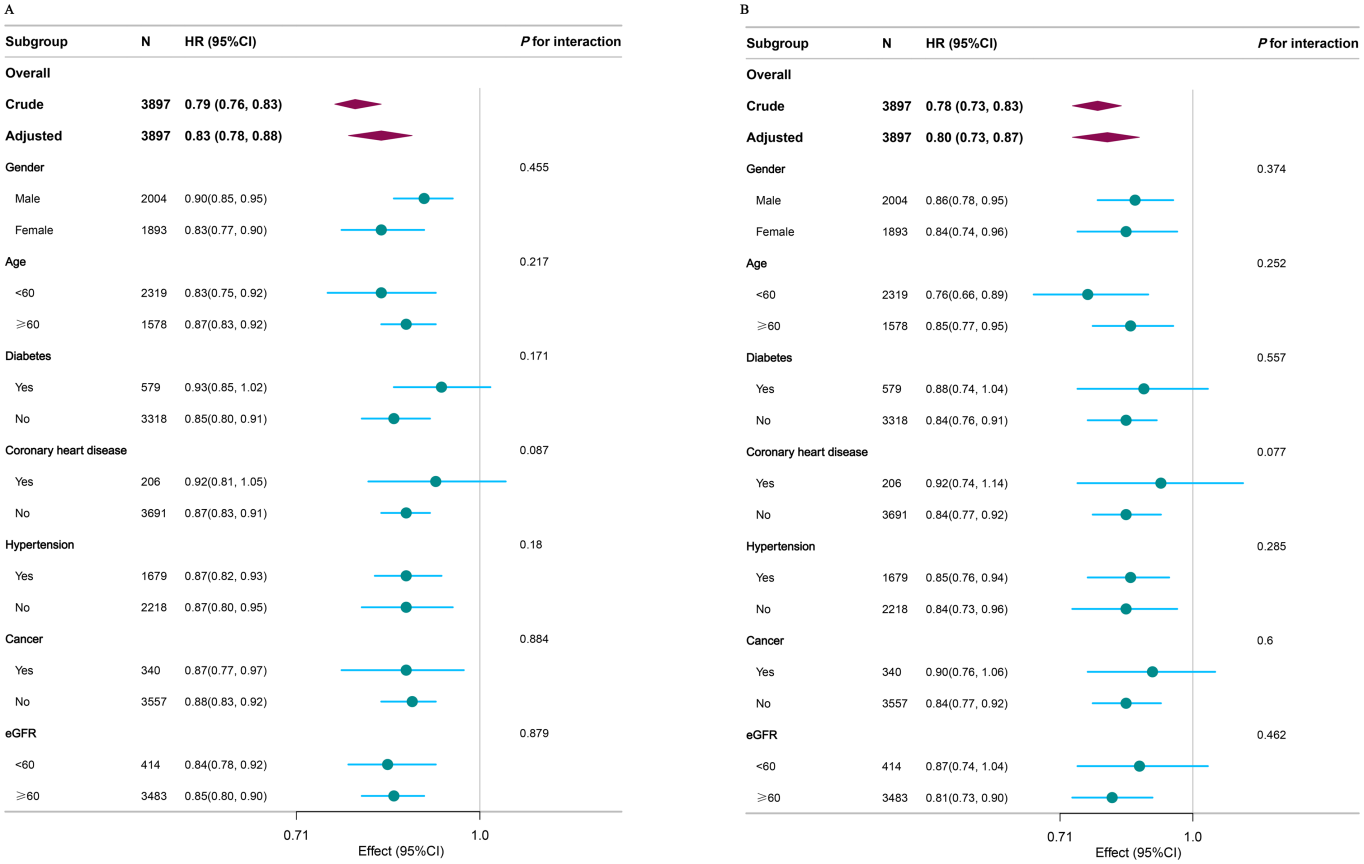
Subgroup analysis of the associations between CCR with all-cause **(A)** and cardiovascular mortality **(B)** among NAFLD. Covariates to be adjusted included gender, age, race, education level, marital status, PIR, moderate activity, smoke status, hypertension, diabetes, coronary heart disease, cancer, BMI, eGFR, HbA1c, UA, HDL, TG, TC, and covariates related to stratification factors were not adjusted. CCR, creatinine to cystatin C ratio; NAFLD, nonalcoholic fatty liver disease; PIR, income to poverty ratio; BMI, body mass index; eGFR, estimated glomerular filtration rate; HbA1c, glycosylated hemoglobin type A1C; UA, uric acid; HDL, hdl-cholesterol; TG, triglycerides; TC, total cholesterol.

Stratified analyses were conducted across various subgroups to evaluate potential effect modifications in the association between CCR and mortality. After adjusting for confounders, no significant interactions were observed across subgroups stratified by gender, age, diabetes, coronary heart disease, hypertension, cancer, or eGFR (Figure 4). To further validate the robustness of our findings, we conducted sensitivity analyses (Supplementary Table 1). After excluding participants with missing covariates, the analysis of the remaining 3,480 individuals consistently demonstrated significant inverse associations between CCR and both all-cause and cardiovascular mortality (all *P* for trend <0.001). These findings, supported by comprehensive subgroup and sensitivity analyses, further confirm the reliability of our results, demonstrating consistent inverse associations between CCR and mortality.

## 4 Discussion

This nationwide study of U.S. adults examined the link between CCR and both all-cause and cardiovascular mortality during long-term follow-up in individuals with NAFLD. Our study identified a notable inverse relationship between CCR and both all-cause and cardiovascular mortality. RCS analysis demonstrated an L-shaped relationship between CCR and all-cause mortality, while the relationship with cardiovascular mortality showed a monotonic decline. The robustness of these findings was further validated through subgroup and sensitivity analyses.

Low muscle mass has detrimental effects on quality of life, economic burden, and healthcare costs (36). A few previous studies have explored the relationship between muscle mass and cardiovascular disease risk and mortality (37, 38). Additionally, several cohort studies have analyzed the association between Cardio-Cerebrovascular Risk and mortality. CCR has been identified as a useful prognostic factor for sarcopenia in esophageal cancer patients, as well as for postoperative complications and long-term survival (39). A retrospective cohort study also found that elevated CCR correlated with improved survival rates in patients undergoing intensive care and continuous kidney replacement therapy (25). Furthermore, liver fat infiltration assessed by the FLI score was significantly associated with cardiovascular disease risk and mortality in patients with newly diagnosed type 2 diabetes (40). Our results align with these findings, showing a significant association between CCR and improved prognosis. However, Our study features a larger sample size, an extended follow-up period, and broadens the association to include all-cause and cardiovascular mortality within the U.S. NAFLD population.

While the exact biological mechanisms linking CCR to mortality with NAFLD remain unclear, various potential mechanisms could be involved. Higher CCR values might protect against NAFLD by improving insulin sensitivity, reducing systemic inflammation, and enhancing glucose metabolism through increased muscle mass. CCR is acknowledged as a proxy biomarker for muscle mass and sarcopenia (19, 41). The rise in CCR indicates enhanced muscle mass, contributing to glucose homeostasis since skeletal muscle is crucial for about 80% of glucose clearance under euglycemic and hyperinsulinemic conditions (42). Additionally, a cohort study found a negative correlation between changes in CCR and blood pressure, hemoglobin A1c, and lipid levels (43). Furthermore, research involving 6,558 participants showed a positive correlation between inflammation markers like hs-CRP and NAFLD (44). A study indicated that as CCR increased across, CRP levels decreased, suggesting that a higher CCR may reflect a lower inflammatory status (26). Therefore, as a straightforward sarcopenia index (18), the connection between CCR and mortality with NAFLD might also encompass these changes, necessitating additional experimental research.

This study also discovered the following points: In our study population, there was a significant gender difference in CCR, which may be related to the normal physiological structural difference, with male muscle content being significantly higher than that of females (45). We further conducted subgroup analysis by gender and performed interaction tests, and the results remained stable. There was a large difference in education levels. Individuals with higher education had higher CCR, while those with a high school education or lower had lower CCR. Income levels showed a similar trend, suggesting that individuals with higher education and higher income tend to have higher muscle content. This may be related to better nutrition, as individuals with higher education and income levels typically have better nutrition, as well as enhanced personal health awareness (46).

This study had several limitations. First, as a cohort study, which is inherently an observational study, it cannot establish causality. Second, despite adjustment for multiple confounders, residual confounding or measurement errors may still affect the results. Additionally, inherent limitations of the FLI, the constraints of single measurements, and the lack of consideration for the impact of medications or other treatments also necessitate a cautious interpretation of the findings.

## 5 Conclusion

In conclusion, our study demonstrates that higher CCR is associated with reduced all-cause and cardiovascular mortality. The calculation of CCR is simple, convenient, and cost-effective, making it a promising indicator of muscle mass. In clinical practice, regular monitoring of CCR can help detect changes in muscle mass early and assist in identifying high-risk patients, optimizing monitoring protocols, and guiding targeted interventions to improve patient outcomes. These applications of CCR can contribute to improved outcomes in patients with NAFLD.

## Data Availability

The datasets presented in this study can be found in online repositories. The names of the repository/repositories and accession number(s) can be found at: Publicly available and de-identified data used in this analysis can be found in the CDC National Center for Health Statistics NHANES database at https://www.cdc.gov/nchs/nhanes/index.htm. The mortality data were obtained from NDI maintained by the Centers for Disease Control and Prevention (accessible at https://www.cdc.gov/nchs/data-linkage/mortality-public.htm). Further inquiries can be directed to the corresponding author.

## Glossary

NAFLD: Non-alcoholic Fatty Liver Disease
CCR: Creatinine-to-cystatin C ratio
NHANES: National Health and Nutrition Examination Survey
NDI: National Death Index
FLI: Fatty liver index
STROBE: guidelines Strengthening the Reporting of Observational Studies in Epidemiology
RCS: Restricted cubic spline
IQR: Interquartile range
PIR: Income to poverty ratio
BMI: Body mass index
eGFR: Estimated glomerular filtration rate
TC: Total cholesterol
HDL: HDL-Cholesterol
TG: Triglycerides
HbA1c: Glycosylated hemoglobin A1C
UA: Uric acid
T: Tertile
SD: Standard deviation
HR: Hazard ratio
CI: Confidence interval
Ref: Reference
