# Correction: Creatinine-to-cystatin C ratio and all-cause and cardiovascular mortality in U.S. adults with nonalcoholic fatty liver disease: a nationwide cohort study

**DOI:** 10.3389/fnut.2025.1641656

**Published:** 2025-06-27

**Authors:** Yuanyuan Chen, Bing Yang, Huihui Chen, Jun Chen, Jinmin Cao, Huijie Wang, Chuantie Chen

**Affiliations:** ^1^Department of Liver Diseases, Shenzhen Third People's Hospital, The Second Affiliated Hospital of Southern University of Science and Technology, National Clinical Research Center for Infectious Diseases, Shenzhen, China; ^2^Department of Gastroenterology, Longgang Central Hospital of Shenzhen, Shenzhen, China; ^3^Guangzhou University of Traditional Chinese Medicine, Guangzhou, China; ^4^Department of Dermatology, Hunan Aerospace Hospital, Changsha, China; ^5^Department of Endoscopy, Shijiazhuang Traditional Chinese Medicine Hospital, Shijiazhuang, China

**Keywords:** creatinine to cystatin C ratio, muscle mass, nonalcoholic fatty liver disease, NHANES, fatty liver index, cohort study, L-shape, mortality

In the published article, there was an error in affiliations 2 and 4. Instead of “^2^Department of Dermatology, Hunan Aerospace Hospital, Changsha, China,” it should be “^2^Department of Gastroenterology, Longgang Central Hospital of Shenzhen, Shenzhen, China.” Instead of “^4^Department of Gastroenterology, Longgang Central Hospital of Shenzhen, Shenzhen, China”, it should be “^4^Department of Dermatology, Hunan Aerospace Hospital, Changsha, China.”

In the published article, there was an error in the correspondence details, the author Yuanyuan Chen was erroneously assigned as the corresponding author. The corrected author list with the corrected correspondence details appears below:

Yuanyuan Chen^1^, Bing Yang^2^, Huihui Chen^3^, Jun Chen^1^, Jinmin Cao^4^, Huijie Wang^5^ and Chuantie Chen^2*^

^*^ **Correspondence:**

Chuantie Chen

guihuotie@163.com

The original article has been updated.

