# Supplementary material for: Creatinine-to-cystatin C ratio and all-cause and cardiovascular mortality in U.S. adults with nonalcoholic fatty liver disease: a nationwide cohort study
Source: Front Nutr. 2025 Jun 3;12:1587757. doi: 10.3389/fnut.2025.1587757 (PMC12172250; doi:10.3389/fnut.2025.1587757)
Supplement: Supplementary file 1 [file Table_1.docx]

**Supplementary Table 1.** The relationships between CCR and mortality with NAFLD (After excluding participants with missing covariates)

| **Variable** | **Total** | **Events** | **Model 1** | | **Model 2** | | **Model 3** | |
| --- | --- | --- | --- | --- | --- | --- | --- | --- |
|  |  |  | **HR (95%CI)** | ***P* value** | **HR (95%CI)** | ***P* value** | **HR (95%CI)** | ***P* value** |
| *All-cause mortality* |  |  |  |  |  |  |  |  |
| CCR | 3480 | 1014 | 0.79 (0.76, 0.83) | <0.001 | 0.84 (0.79, 0.89) | <0.001 | 0.83 (0.77, 0.88) | <0.001 |
| CCR tertile |  |  |  |  |  |  |  |  |
| T1 | 1158 | 468 | 1(Ref) |  | 1(Ref) |  | 1(Ref) |  |
| T2 | 1162 | 330 | 0.58 (0.49, 0.68) | <0.001 | 0.70 (0.58, 0.85) | <0.001 | 0.67 (0.54, 0.84) | <0.001 |
| T3 | 1160 | 216 | 0.32 (0.26, 0.40) | <0.001 | 0.49 (0.35, 0.68) | <0.001 | 0.46 (0.33, 0.65) | <0.001 |
| *P* for trend |  |  |  | <0.001 |  | <0.001 |  | <0.001 |
| *Cardiovascular mortality* |  |  |  |  |  |  |  |  |
| CCR | 3480 | 287 | 0.79 (0.74, 0.84) | <0.001 | 0.81 (0.73, 0.90) | <0.001 | 0.81 (0.73, 0.90) | <0.001 |
| CCR tertile |  |  |  |  |  |  |  |  |
| T1 | 1158 | 130 | 1(Ref) |  | 1(Ref) |  | 1(Ref) |  |
| T2 | 1162 | 95 | 0.68 (0.52, 0.89) | 0.005 | 0.76 (0.54, 1.05) | 0.098 | 0.77 (0.53, 1.10) | 0.153 |
| T3 | 1160 | 62 | 0.30 (0.21, 0.43) | <0.001 | 0.40 0.25, 0.65 | <0.001 | 0.40 (0.24, 0.68) | <0.001 |
| *P* for trend |  |  |  | <0.001 |  | <0.001 |  | <0.001 |

Model 1 unadjusted

Model 2 adjusted for gender, age and race

Model 3 further adjusted for education level, marital status, PIR, moderate activity, smoke status, hypertension, diabetes, coronary heart disease, cancer, BMI, eGFR, HbA1c, UA, HDL, TG and TC based on Model 2.

Abbreviations: CCR, creatinine to cystatin C ratio; NAFLD, nonalcoholic fatty liver disease; T, tertile; HR, hazard ratio; CI, confidence interval; Ref, reference; PIR, income to poverty ratio; BMI, body mass index; eGFR, estimated glomerular fltration rate; HbA1c, glycosylated hemoglobin type A1C; UA, uric acid; HDL, hdl-cholesterol; TG, triglycerides; TC, total cholesterol.
